# *ZKSCAN1* gene and its related circular RNA (circ*ZKSCAN1*) both inhibit hepatocellular carcinoma cell growth, migration, and invasion but through different signaling pathways

**DOI:** 10.1002/1878-0261.12045

**Published:** 2017-03-17

**Authors:** Zhicheng Yao, Jingyan Luo, Kunpeng Hu, Jizong Lin, He Huang, Qiangliang Wang, Peng Zhang, Zhiyong Xiong, Chonghua He, Zejian Huang, Bo Liu, Yang Yang

**Affiliations:** ^1^ Department of General Surgery The Third Affiliated Hospital of Sun Yat‐sen University Guangzhou Guangdong China; ^2^ R&D Unit 602 Forevergen Biosciences Center Guangzhou Guangdong China; ^3^ Department of Hepatobiliary Surgery Sun Yat‐sen Memorial Hospital Sun Yat‐sen University Guangzhou Guangdong China; ^4^ Department of Liver Transplantation The Third Affiliated Hospital of Sun Yat‐sen University Guangzhou Guangdong China

**Keywords:** circular RNA, HCC, qRT‐PCR, RNA‐seq, ZKSCAN1

## Abstract

There is increasing evidence that circular RNA (circRNA) are involved in cancer development, but the regulation and function of human circRNA remain largely unknown. In this study, we demonstrated that *ZKSCAN1*, a zinc finger family gene, is expressed in both linear and circular (*circZKSCAN1*) forms of RNA in human hepatocellular carcinoma (HCC) tissues and cell lines. Here, we analyzed a cohort of 102 patients and found that expression of both *ZKSCAN1* mRNA and *circZKSCAN1* was significantly lower (*P *< 0.05) in the HCC samples compared with that in matched adjacent nontumorous tissues by reverse transcription PCR (RT‐PCR). The low expression level of *ZKSCAN1* was only associated with tumor size (*P *=* *0.032), while the *cirZKSCAN1* levels varied in patients with different tumor numbers (*P *<* *0.01), cirrhosis (*P *=* *0.031), vascular invasion (*P *=* *0.002), or microscopic vascular invasion (*P *=* *0.002), as well as with the tumor grade (*P *<* *0.001). Silencing both *ZKSCAN1* mRNA and *circZKSCAN1* promoted cell proliferation, migration, and invasion. In contrast, overexpression of both forms of RNA repressed HCC progression *in vivo* and *in vitro*. Silencing or overexpression of both forms of RNA did not interfere with each other. RNA‐seq revealed a very different molecular basis for the observed effects; *ZKSCAN1* mRNA mainly regulated cellular metabolism, while *circZKSCAN1* mediated several cancer‐related signaling pathways, suggesting a nonredundant role for *ZKSCAN1* mRNA and circRNA. In conclusion, our results revealed two post‐translational products (*ZKSCAN1* mRNA and *circZKSCAN1*) that cooperated closely with one another to inhibit growth, migration, and invasion of HCC. *cirZKSCAN1* might be a useful marker for the diagnosis of HCC.

**Abbreviations**
AASLD: American Association for the Study of Liver Diseases
AFP: alpha‐fetoprotein
circRNA: circular RNA
HCC: human hepatocellular carcinoma
hESCs: H1 human embryonic stem cells
MVI: microscopic vascular invasion
NCCN: National Comprehensive Cancer Network
RPKM: the reads per kilobase of exon model per million mapped reads
RT‐PCR: reverse transcription PCR

## 1 Introduction

Hepatocellular carcinoma (HCC) is one of the most common malignancies worldwide. Statistics have shown that approximately 740 000 people are newly diagnosed with HCC and that this disease accounts for approximately 700 000 deaths annually worldwide (Marquardt *et al*., 2015). Approximately 55% of newly diagnosed patients were found in China, which consequently was regarded as the area with the highest prevalence of HCC (Torre *et al*., 2015). With the improvement of diagnostic approaches and treatment strategies, patients with HCC can be detected at an early stage and subjected to radical surgeries with a favorable prognosis. However, many patients (approximately 70% of newly diagnosed patients) with HCC missed using current diagnostic standards, and therefore, the best surgical opportunities were missed. Traditionally, alpha‐fetoprotein (AFP) has been widely used for the clinical diagnosis of HCC. However, as a biomarker, AFP has limited clinical diagnostic value (especially for early diagnosis). Additionally, the 2010 guidelines of the American Association for the Study of Liver Diseases no longer recommend AFP for screening (Lin *et al*., 2015). To improve the therapeutic effect and prognosis of patients with HCC, it is imperative to search for more efficient biomarkers to increase the rate of early diagnosis of HCC. Therefore, a better understanding of the potential molecular mechanism underlying the occurrence and development of liver cancer, searches for new diagnostic markers and new targets of clinical treatment are of great important in liver cancer research.

Circular RNA (circRNA) are a special class of noncoding RNA that feature a covalently closed continuous loops (Jens, 2013). Compared with linear RNA, circRNA have the remarkable characteristic of noncanonical splicing without a free 3′ or 5′ end (Hentze and Preiss, 2013). circRNA were reported more than 20 years ago (Nigro *et al*., 1991), but have mostly been misinterpreted as splicing errors. Not until 2012 were circRNA rediscovered based on RNA sequencing (RNA‐seq) data and shown to be widespread and diverse in eukaryotic cells. More than 20 000 different circRNA species have been reported in human and mouse tissues, and 700 in *Caenorhabditis elegans*. Typically, circular isoforms account for 5–10% of the total number of transcripts of their corresponding coding gene, but certain circRNA are up to 200 times more abundant than their linear counterparts (Hansen *et al*., 2013; Jeck *et al*., 2013; Salzman *et al*., 2013). Concerning their function, it is speculated that circRNA serve as epigenetic microRNA (miRNA) sponges (Hansen *et al*., 2013). In fact, previous studies have already validated this hypothesis (Hansen *et al*., 2013; Lasda and Parker, 2014). Recently, researchers have found that circRNA are involved in the development of several types of diseases, such as atherosclerosis and nervous system disorders (Burd *et al*., 2010; Chen and Old, 1990). Moreover, circRNA are becoming a new research hotspot in various cancers. For example, Hsa_circ_0001649 and hsa_circ_0005075 have been reported as potential diagnostic biomarkers for HCC, but how they regulate cancer progression at a mechanistic level is unclear (Qin *et al*., 2015). Additional studies are needed to investigate the role of circRNA and their potential diagnostic value in HCC.

A zinc finger with KRAB and SCAN domains 1 (*ZKSCAN1*, also named ZNF139) is a member of the zinc finger protein family. Members of the zinc finger protein family play regulatory roles for a variety of genes at the transcriptional level, have important effects on the maintenance of normal life activities, and are closely related to tumorigenesis, development, metastasis, and drug tolerance. van Dekken *et al*. (2009) found that increased *ZKSCAN1* expression in adenocarcinoma at the esophagogastric junction was related to the proliferation of tumor cells. The ZKSCAN1 protein is overexpressed in certain gastroesophageal cancers (van Dekken *et al*., 2009) and has been suggested to regulate GABA type A receptor expression in the brain (Mulligan *et al*., 2012). However, to date, there have been no reports examining the relationship between *ZKSCAN1* and HCC. Deep sequencing of the transcriptomes of numerous human cell lines, including HEK293, HeLa, and H1 human embryonic stem cells (hESCs), has revealed exon–exon junction reads connecting the 3′ end of exon 3 to the 5′ end of exon 2 (Jeck *et al*., 2013; Salzman *et al*., 2013). This finding suggests that exons 2 and 3 of *ZKSCAN1* may be spliced together to form a covalently linked 668‐nt circular RNA termed *circZKSCAN1* (circBase ID: hsa_circ_0001727). *circZKSCAN1* is particularly abundant in human brain and liver (Liang and Wilusz, 2014); however, its function in the regulation of HCC remains unknown.

In the present study, the role of *ZKSCAN1* gene and its relative circRNA (*circZKSCAN1*) in the regulation of hepatocellular carcinoma cell growth, migration, and invasion and the potential mechanism were investigated.

## 2 Materials and methods

### 2.1 Patients and tissue specimens

A total of 102 hepatocellular carcinoma tissue specimens and paired adjacent nontumorous tissues were collected from the hepatocellular carcinoma surgical specimens from February 2015 to February 2016 at The Second and The Third Affiliated Hospital of Sun Yat‐sen University, China. After dissection, all tissue specimens were immediately preserved in RNA‐fixer Reagent (Bioteke, Beijing, China) and stored at −80 °C until use. The corresponding adjacent nontumorous tissues were collected 5 cm from the edge of the cancer, as evaluated by an experienced pathologist. The histological grade was assessed following the National Comprehensive Cancer Network (NCCN) Clinical Practice Oncology Guidelines (V.1.2011). No radiotherapy, chemotherapy, or targeted therapy was conducted prior to surgery. All human studies were approved by The Institute Research Medical Ethics Committee of Sun Yat‐Sen University. Written informed consent was obtained from all subjects.

### 2.2 Cell culture and lentivirus‐mediated stable cell line construction

The human normal liver L02 cell line and the HCC cell lines (Huh7, SMMC‐7721, BEL‐7402, HepG2, and Hep3B) were purchased from Sun Yat‐Sen University Laboratory Animal Center and maintained in Dulbecco's modified Eagle's medium (DMEM) with 10% (v/v) FBS (Invitrogen, Carlsbad, CA, USA). The cell lines were maintained in a humidified chamber with 5% CO_2_ at 37 °C.

Overexpression of *circZKSCAN1* was achieved according to a previous study (Liang and Wilusz, 2014). Dongming Liang reported that miniature introns containing the splice sites, along with short (~ 30‐ to 40‐nucleotide) inverted repeats, are sufficient to allow the intervening exons of *ZKSCAN1* to circularize in cells. Consequently, we synthesized the full‐length circ*ZKSCAN1* together with its inverted repeat sequence, according to a previous report (Liang and Wilusz, 2014), and cloned it into the lentiviral vector LV003 (Forevergen Biosciences, Guangzhou, China). The CDS sequence of *ZKSCAN1* (NM_003439.2) was synthesized and cloned into the lentiviral vector LV003 (Forevergen Bioscience Center). The gene was synthesized by Shanghai Generay Biotech Co. For the knockdown experiments, the siRNA target sequences were as follows: si‐circ: 5′‐CAGUCACGAGGAAUAGUAA‐3′; si‐linear: 5′‐ACCUCGGAAGAUUCAGCAU‐3′. The shRNA expression cassettes containing the sense‐loop (TTCAAGAGA)‐antisense‐termination signal T6 were inserted downstream of the U6 promoter in the LV008 vector (Forevergen Biosciences). Negative control (NC) expression cassettes (sequence 5′‐CTTTCTCCGAACGTGTCAC‐3′) were used for the control cell line. The expression vectors were mixed with plasmids pGag/Pol, pRev, and pVSV‐G and transfected into 293T cells using Lipofectamine 2000. The supernatant was collected after 48 and 72 h, and the infections were conducted in the presence of 5–10 μg·mL^−1^ Polybrene. HepG2 and SMMC‐7721 stable cells were selected with 2 μg·mL^−1^ puromycin after transduction.

### 2.3 RT‐qPCR and RNase R treatment

Total RNA was isolated from cultured cells and fresh tissues using TRIzol RNA isolation reagent (Life Technologies, Carlsbad, CA, USA) according to the manufacturer's instructions. The RNA integrity and concentration were determined using the Agilent RNA 6000 Nano Kit and Agilent 2100 Bioanalyzer (Agilent, Santa Clara, CA, USA). Reverse transcription was performed using M‐MLV reverse transcriptase (Promega, Madison, WI, USA) and random primers. The primers were utilized as described in Table 1. All qPCR were performed using the GoTaq^®^ qPCR Master Mix kit (Promega) according to the manufacturer's protocol. The threshold cycle (*C*_t_) value for each sample was calculated using the ABI analytical thermal cycler, and the relative RNA level was normalized to the GAPDH mRNA value. RNA expression data were calculated using the ΔΔ*C*_t_ method. Moreover, the RNase R digestion reaction was performed as previously reported (Jeck *et al*., 2013). Total RNA (5 μg) was incubated for 15 min at 37 °C with or without 3 U·μg^−1^ of RNase R (Epicentre Biotechnologies, Madison, WI, USA). The RNA was subsequently purified by phenol–chloroform extraction and then subjected to RT‐qPCR.

**Table 1:** Primers used in this study

Primer names	Sequences (5′→3′)
Hsacirc*ZKSCAN1*_F divergent	AGTCCCACTTCAAACATTCGTCT
Hsacirc*ZKSCAN1*_R divergent	CACCTTCACTATTACGATACCATCC
Hsacirc*ZKSCAN1*_F convergent	TACCGCCCCGATAGTGGAGA
Hsacirc*ZKSCAN1*_R convergent	TGAAGTGGGACTGGGTGGC
Hsa*ZKSCAN1* F	TGTAATGAGTGCGGGAAGG
Hsa*ZKSCAN1* R	AATCAGGTATGAGTTTCGGTTG
HsaGAPDH convergent_F	GAGTCAACGGATTTGGTCGT
HsaGAPDH convergent_R	GACAAGCTTCCCGTTCTCAG
HsaGAPDH divergent_F	TCCTCACAGTTGCCATGTAGACCC
HsaGAPDH divergent_R	TGCGGGCTCAATTTATAGAAACCGGG

### 2.4 Western blotting

Western blotting was performed according to standard methods as described previously (Luo *et al*., 2015) using anti‐ZKSCAN1 Abcam (Cambridge, MA, USA) and anti‐GAPDH antibodies Santa Cruz (Santa Cruz, CA, USA). After washing, the membranes were incubated with horseradish peroxidase‐conjugated goat anti‐mouse or anti‐rabbit secondary antibodies (Jackson ImmunoResearch, West Grove, PA, USA) and visualized using enhanced chemiluminescence reagents (Forevergen Biosciences).

### 2.5 Cell proliferation, migration, and invasion assay

The [3‐(4,5‐dimethylthiazol‐2‐yl)‐5‐(3‐carboxymethoxyphenyl)‐2‐(4‐sulfophenyl)‐2H‐tetrazolium] MTS viability assay (Promega) was performed according to standard methods as described previously (Luo *et al*., 2015). *ZKSCAN1* and *circZKSCAN1* knockdown or overexpression cell lines and control cells were seeded at 2 × 10^4^ cells in 100 μL medium per well of a 96‐well plate Corning Incorporated (Corning, NY, USA) for the MTS assay (Qi *et al*., 2014; Qin *et al*., 2016). The cells were incubated for a total of 3 days with fresh culture medium. Proliferation was analyzed using the MTS reagent at day 1, day 2, and day 3. Triplicate samples were evaluated for each treatment.

For the invasion assay, the transwell system and Matrigel BD Biosciences (New York, NY, USA) were used according to the manufacturers’ protocols and the invasion assay was performed according to the methods as described previously (Qi *et al*., 2014; Qin *et al*., 2016). Aliquots of 1 × 10^6^ cells were seeded into the upper chambers, which were precoated with Matrigel, and cultured in serum‐free DMEM. The lower compartment was filled with DMEM supplemented with 10% FBS as a chemoattractant. After incubation for 48 h, the cells remaining in the upper chamber were removed, and the cells at the bottom of the insert were fixed, stained with 0.5% crystal violet, and counted under a microscope (Olympus Corp., Tokyo, Japan). The results from three independent experiments were averaged. For the migration assay, the cells were seeded into the upper chambers without a Matrigel coating. The rest of the assay was conducted as described for the invasion assay. Cell numbers in five high‐power fields were counted after crystal violet staining. Three biological replicates were conducted.

### 2.6 Xenografts in mice

Twenty‐five (*n* = 5 per group) 6‐week‐old male nude mice were purchased from Sun Yat‐Sen University Laboratory Animal Center. Control (negative cells) and treated cells (cells transduced with the indicated lentivirus vector) were diluted to a concentration of 1 × 10^6 ^mL^−1^ in physiological saline. Mice were injected subcutaneously with 0.1 mL of the suspension into the back flank. When a tumor was palpable, tumor growth was measured every week with a caliper. After five weeks, the mice were killed, and the tumors were dissected and weighed. The tumor volume (V) was calculated according to the following formula: *V* = *L* × W2 × 0.5 (L, length; W, width). All animal studies were approved by The Institute Research Medical Ethics Committee of Sun Yat‐Sen University.

### 2.7 Immunohistochemistry and fluorescence *in situ* hybridization (FISH)

Immunohistochemistry was performed according to the standard methods as described previously (Yi *et al*., 2016). Briefly, paraffin‐embedded tissues were stained with anti‐ZKSCAN1 antibody (1 : 100 dilution; Abcam) at 4 °C overnight. Rabbit IgG was used as a negative control. After washing, the slides were treated with rabbit biotinylated secondary antibody at room temperature for 30 min. The slides were then stained with the ABC Elite kit Vector Labs (Burlingame, CA, USA). Finally, the slides were counterstained with hematoxylin, dehydrated, cleared, and then mounted with Permount mounting medium Fisher Scientific (Fair Lawn, NY, USA).

For the FISH assay, PCR fragments containing the T7 promoter were amplified using specific primers for the back splice region of *circZKSCAN1*. RNA probes were transcribed using the TranscriptAid T7 High Yield Transcription Kit (Thermo Scientific, Rochester, NY, USA) with the corresponding PCR product as a template for transcription, and they were labeled with Alexa Fluor 546 using the ULYSIS Nucleic Acid Labeling Kit (Invitrogen) according to the manufacturer's protocol. HepG2 cells were grown to 50–75% confluence at the time of fixation. After prehybridization, the cells were hybridized to Alexa Fluor 546‐labeled probes specific to *circZKSCAN1* at 60 °C overnight. Signals were detected under a microscope (Olympus Corp.). Primers for FISH analysis were Hsacirc cZKSCAN1_P1‐FISH: TAATACGACTCACTATAGGGAGTCCCACTTCAAACATTCGTCT, HsacircZKSCAN1_P2‐FISH: CACCTTCACTATTACGATACCATCC.

### 2.8 RNA‐seq

Following RNA isolation, the RNA was quantified (Qubit RNA Assay Kit; Life Technologies, Inc.), and the quality was assessed (RNA6000 Nano Kit and BioAnalyzer 2100; Agilent). Next, 1000 ng was used as the input for the VAHTSTM mRNA‐seq v2 Library Prep Kit for Illumina^®^ (Vazyme, Inc., Piscataway, NJ, USA), and sequencing libraries were created according to the manufacturer's protocol. Briefly, poly(A) RNA was purified via two rounds of hybridization to Dynal Oligo (dT) beads. Poly(A)+ RNA was fragmented and then used for first‐ and second‐strand cDNA synthesis with random hexamer primers. The cDNA fragments were treated with the End‐It DNA EndRepair Kit to repair the ends, then modified with Klenow to add an A at the 3′ end of the DNA fragments, and finally ligated to the adapters. The ligated cDNA products were subjected to PCR amplification. The library quality was determined using a Bioanalyzer 2100 (Agilent). The RNA‐seq libraries were sequenced using the Illumina HiSeq 4000 platform. Reads were aligned to the human reference genome GRCh37/hg19 using tophat v2.1.0 (Langmead, 2012). The reads per kilobase of exon model per million mapped reads (RPKM) of each gene was calculated based on the length of the gene and the read counts mapped to the gene. Differential expression analysis of two conditions/groups (two biological replicates per condition) was performed using the previously described statistical model (Audic and Claverie, 1997). The resulting *P*‐values were adjusted using Benjamini and Hochberg's approach for controlling the false discovery rate. Genes with an adjusted *P*‐value < 0.05 were considered differentially expressed. We used kobas software (Peking University, Beijing, China) to test the statistical enrichment of the differentially expressed genes in the KEGG pathway. circRNA were used as seeds to enrich the circRNA–miRNA–gene network according to the analysis of miRanda (http://www.microrna.org/) combined with our RNA‐seq data. Cytoscape (http://www.cytoscape.org/) was applied to build a circRNA–miRNA–mRNA interaction network, according to a previous report (Shang *et al*., 2016).

### 2.9 Statistics

Data are presented as the mean ± SE. Statistical analyses were conducted with the spss 11.5 statistical software package. The primary statistical test was one‐way ANOVA. Differences in the expression of *ZKSCAN1* and *cirZKSCAN1* between different clinical parameters of HCC were analyzed using the Student's *t*‐test. *P* < 0.05 was considered statistically significant. The receiver‐operating characteristic (ROC) is a comprehensive index that is used to reflect the sensitivity and specificity of continuous variables. A larger area under the curve reflects a higher diagnostic value of the variable. To evaluate whether *ZKSCAN1* and *circZKSCAN1* could serve as a potential marker for the diagnosis of HCC, the ROC curve was constructed, and the AUC value was calculated to explore the diagnostic value of *ZKSCAN1* and *circZKSCAN1*.

## 3 Results

### 3.1 *ZKSCAN1* was down‐regulated in human HCC and a circular form of its RNA, *circZKSCAN1*, was validated

**Figure 1:**
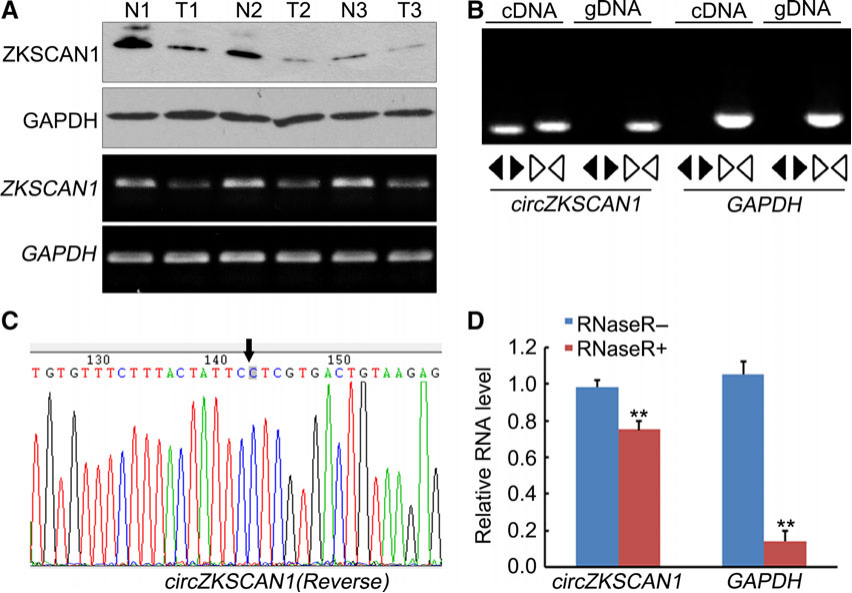
The mRNA and protein expression levels of *ZKSCAN1*, and *circZKSCAN1* validation in human HCC tissues. (A) Western blot and RT‐PCR analysis of the expression level of ZKSCAN1 in three randomly selected hepatocellular carcinoma specimens and the paired adjacent nontumorous tissues. GAPDH was used as the reference in both analyses. N: adjacent nontumorous tissue; T: hepatocellular carcinoma tissue. (B) Divergent primers were used to amplify circular RNA in cDNA but not genomic DNA (gDNA). Convergent primers can amplify both circular RNA and linear RNA, GAPDH, and the linear control. (C) Sanger sequencing of a PCR product resulting from divergent primers demonstrating the head‐to‐tail splicing of this exon. (D) The predicted circular RNA is resistant to RNase R treatment. **P <0.01, in contrast to the linear control gene GAPDH, the circular RNA was resistant to RNase R treatment.

To investigate the expression patterns of *ZKSCAN1*, HCC tissues and adjacent nontumorous tissues were subjected to reverse transcription PCR (RT‐PCR) and western blot analysis, and the results are shown in Fig. 1A. Both the mRNA and protein of *ZKSCAN1* were significantly down‐regulated, indicating that the *ZKSCAN1* gene may be involved in the progression of HCC. RT‐PCR assays were used to verify this circular form of *ZKSCAN1*. The assays performed with divergent primers indicated that *circZKSCAN1* was expressed in the samples of HCC tissues (Fig. 1B). Moreover, Sanger sequencing of the RT‐PCR products amplified by divergent primers further confirmed the back splice junction of *circZKSCAN1* (Fig. 1C). To further confirm the circular characteristics of *cirZKSCAN1*, we used the enzyme RNase R, a highly processive 3′ to 5′ exoribonuclease that does not act on circular RNA (Vincent and Deutscher, 2009). As expected, in contrast to the linear control gene GAPDH, the circular RNA was resistant to RNase R treatment (Fig. 1D), demonstrating that in addition to the *ZKSCAN1* mRNA, there was a naturally circular RNA form of the *ZKSCAN1* gene.

### 3.2 The expression and potential diagnostic values of *ZKSCAN1* and *cirZKSCAN1* in human HCC

**Figure 2:**
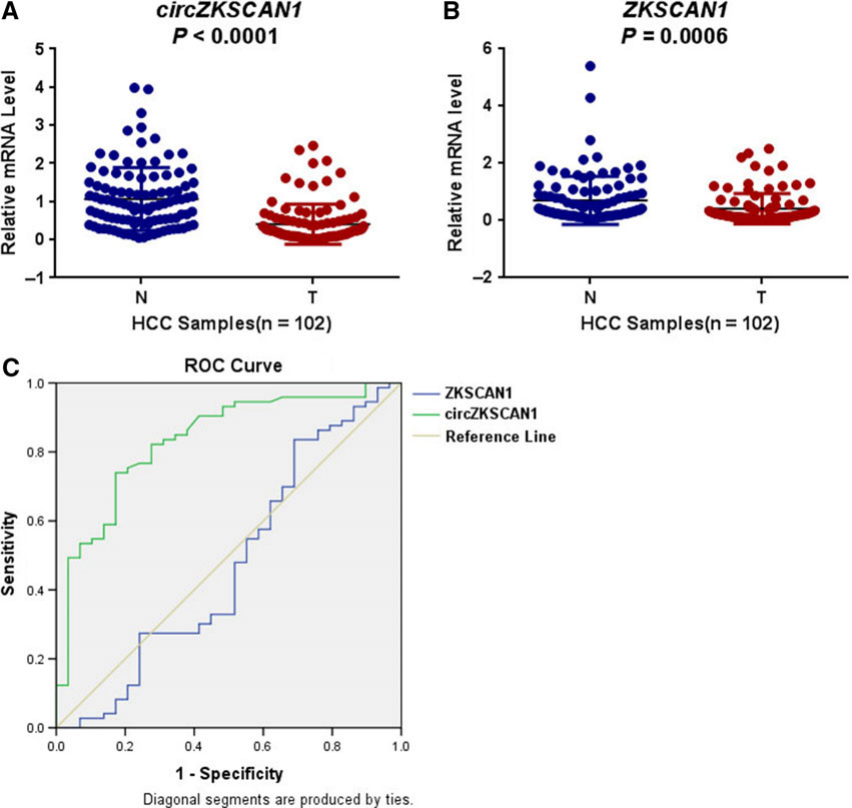
qRT‐PCR analysis of the expression of *ZKSCAN1* and *circZKSCAN1* and the ROC curves of *ZKSCAN1* and *circZKSCAN1*. (A) The expression levels of *circZKSCAN1* in each patient were significantly lower than those in the corresponding adjacent nontumorous tissues. *P *<* *0.001. N: adjacent nontumorous tissue; T: hepatocellular carcinoma tissue. (B) The expression levels of *ZKSCAN1* in each patient were significantly lower than those in the corresponding nontumorous tissues. *P <* *0.05. N: adjacent nontumorous tissue; T: hepatocellular carcinoma tissue. (C) Comparisons of the ROC curves of *circZKSCAN1*,*ZKSCAN1*.

In this study, differential expression of *ZKSCAN1* and *cirZKSCAN1* was observed based on qRT‐PCR analysis of 102 HCC tissue samples and paired adjacent nontumorous liver tissues. The GAPDH gene served as the internal standard. Both linear *ZKSCAN1* and *circZKSCAN1* were significantly down‐regulated in HCC samples (Fig. 2A,B). Further determination of the relationship between the expression of *ZKSCAN1*,* cirZKSCAN1*, and the clinical parameters of HCC showed that *ZKSCAN1* and *cirZKSCAN1* expression was associated with several clinicopathological features. As shown in Table 2, of all the clinical parameters, a low expression level of *ZKSCAN1* was only associated with a tumor size less than 5 cm (*P *=* *0.032). Additionally, the *cirZKSCAN1* levels varied in patients with different tumor numbers (*P *<* *0.01), cirrhosis (*P *=* *0.031), vascular invasion (*P *=* *0.002), or microscopic vascular invasion (MVI, *P *=* *0.002), as well as with the tumor grade (*P *<* *0.001). As shown in Fig. 2C, the area under the ROC curve (AUC) of *cirZKSCAN1* was 0.834, with a sensitivity of 82.2% and specificity of 72.4%, which is much higher than the mRNA of *ZKSCAN1* (AUC = 0.474) (Fig. 2C), indicating that *circZKSCAN1* expression could serve as a biomarker for distinguishing cancerous tissue from adjacent noncancerous liver tissue.

**Table 2:** Baseline demographic and clinical characteristics of the study populations

Characteristics	Patient number	*ZKSCAN1*		*circZKSCAN1*	
		Mean ± SD	*P‐*value	Mean ± SD	*P‐*value
Gender					
Male	87	0.395 ± 0.054	0.390	0.535 ± 0.092	0.960
Female	15	0.523 ± 0.173		0.524 ± 0.145	
Age					
< 60 years	71	0.389 ± 0.060	0.484	0.512 ± 0.080	0.695
≥ 60 years	31	0.470 ± 0.108		0.582 ± 0.196	
HCC_history					
Negative	94	0.414 ± 0.056	0.951	0.537 ± 0.087	0.871
Positive	8	0.402 ± 0.144		0.488 ± 0.176	
Cirrhosis					
Negative	39	0.351 ± 0.063	0.353	0.804 ± 0.191	0.031*
Positive	63	0.452 ± 0.076		0.366 ± 0.049	
HBsAg					
Positive	85	0.434 ± 0.062	0.382	0.449 ± 0.071	0.140
Negative	17	0.310 ± 0.069		0.958 ± 0.323	
AFP1					
< 20	40	0.398 ± 0.078	0.810	0.551 ± 0.095	0.865
≥ 20	62	0.424 ± 0.071		0.522 ± 0.119	
Tumor number					
1	66	0.423 ± 0.069	0.808	0.701 ± 0.118	< 0.001*
> 1	36	0.396 ± 0.079		0.227 ± 0.049	
Tumor size					
< 5 cm	29	0.257 ± 0.075	0.032*	0.647 ± 0.197	0.379
≥ 5 cm	73	0. 476 ± 0.066		0.488 ± 0.082	
Membrane					
Negative	57	0.488 ± 0.077	0.102	0.622 ± 0.127	0.220
Positive	45	0.319 ± 0.068		0.421 ± 0.087	
Vascular invasion					
Negative	74	0.431 ± 0.061	0.594	0.641 ± 0.107	0.002*
Positive	28	0.368 ± 0.105		0.250 ± 0.061	
MVI					
Negative	80	0.367 ± 0.056	0.092	0.616 ± 0.100	0.002*
Positive	22	0.583 ± 0.134		0.232 ± 0.069	
Differentiation					
Moderate‐low + low	27	0.466 ± 0.119	0.553	0.466 ± 0.114	0.615
High + moderate	75	0.395 ± 0.058		0.558 ± 0.103	
Grade					
I + II	73	0.360 ± 0.050	0.193	0.684 ± 0.107	< 0.001*
III + IV	29	0.549 ± 0.134		0.155 ± 0.052	

Notes: Statistical data were analyzed using spss 16.0 software (SPSS, Chicago, IL, USA). *P *<* *0.05 was considered statistically significant, *means *P *<* *0.05.

### 3.3 *ZKSCAN1* and *cirZKSCAN1* were down‐regulated in HCC cell lines and did not affect one another's expression

**Figure 3:**
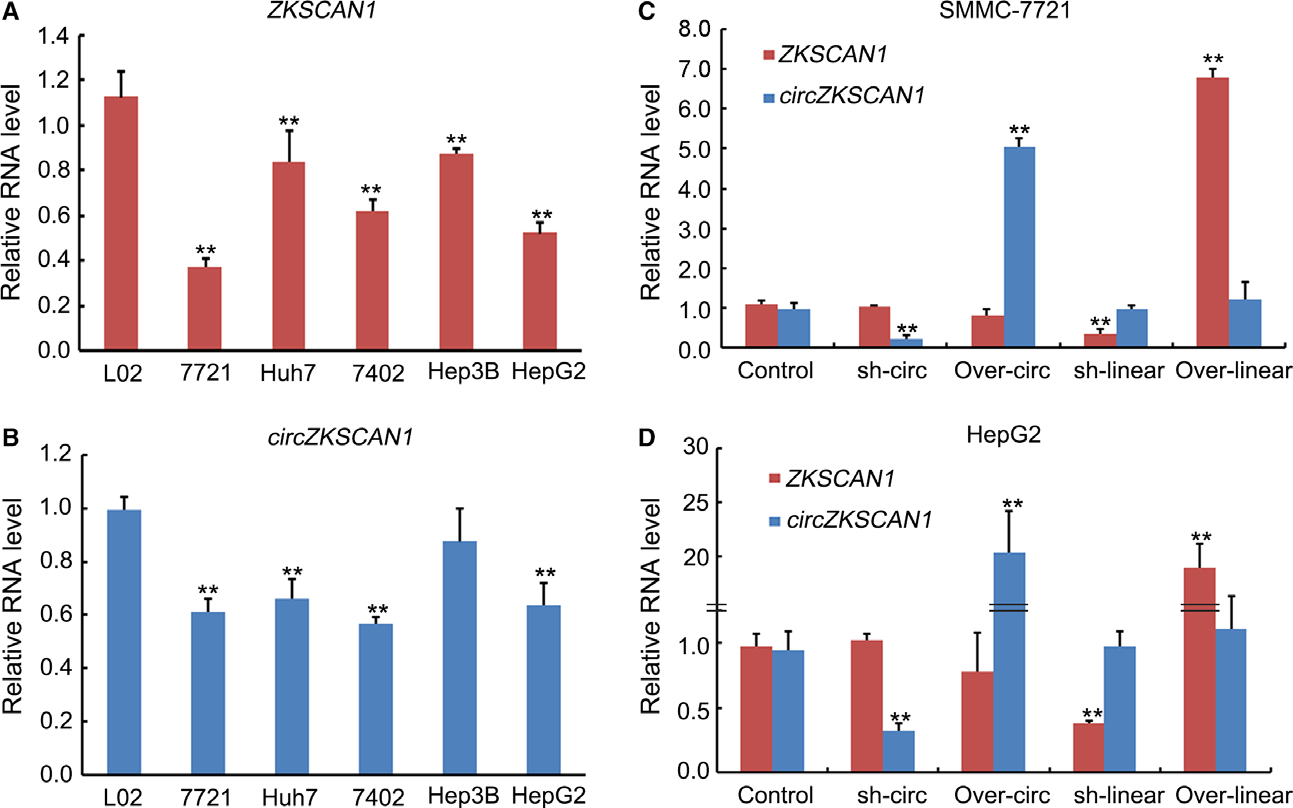
qRT‐PCR analysis of the expression of *ZKSCAN1* and *circZKSCAN1* with and/or without interferences in human HCC cell lines. (A) The expression levels of *ZKSCAN1* in five HCC cell lines (Huh7 cell line, SMMC‐7721 cell line, BEL‐7402 cell line, HepG2 cell line, and Hep3B cell line) were significantly lower than those in the human normal hepatic L02 cell line. **P <* *0.001. (B) The expression levels of *circZKSCAN1* in five HCC cell lines (Huh7 cell line, SMMC‐7721 cell line, BEL‐7402 cell line, Hep3B cell line, and HepG2 cell line) were significantly lower than those in the L02 cell line. **P <* *0.001. (C) The expression levels of *ZKSCAN1* and *circZKSCAN1* in the control, overexpression, and knockdown of *circZKSCAN1* or *ZKSCAN1* SMMC‐7721 cell line. (D) The expression levels of *ZKSCAN1* and *circZKSCAN1* in the control, overexpression, and knockdown of *circZKSCAN1* or *ZKSCAN1* in the HepG2 cell line.

To analyze the potential function of *ZKSCAN1* and *cirZKSCAN1* in HCC cells, we next investigated *ZKSCAN1* mRNA and *cirZKSCAN1* expression in some human HCC cell lines. Five HCC cell lines (Huh7, SMMC‐7721, BEL‐7402, HepG2, and Hep3B) and a control group (human normal liver cell line, L02) were assessed by qRT‐PCR. As shown in Fig. 3A,B, the *ZKSCAN1* mRNA and *cirZKSCAN1* transcripts were down‐regulated in all HCC cell lines compared with the control group. To further investigate the role of *ZKSCAN1* in HCC, we chose two cell lines, HepG2 and SMMC‐7721, which demonstrated the lowest expression levels of both forms of *ZKSCAN1* RNA, to establish stable cell lines. We confirmed the efficient silencing and ectopic expression by qRT‐PCR. In addition, our results indicated that overexpression or knockdown treatments of *ZKSCAN1* or *cirZKSCAN1* could effectively increase or decrease their mRNA expression levels but did not interfere with their interaction with one another in SMMC‐7721 cells and HepG2 cells (Fig. 3C,D).

### 3.4 Influence of overexpression and knockdown of *ZKSCAN1* and *cirZKSCAN1* on proliferation, migration, and invasion of HCC cells

**Figure 4:**
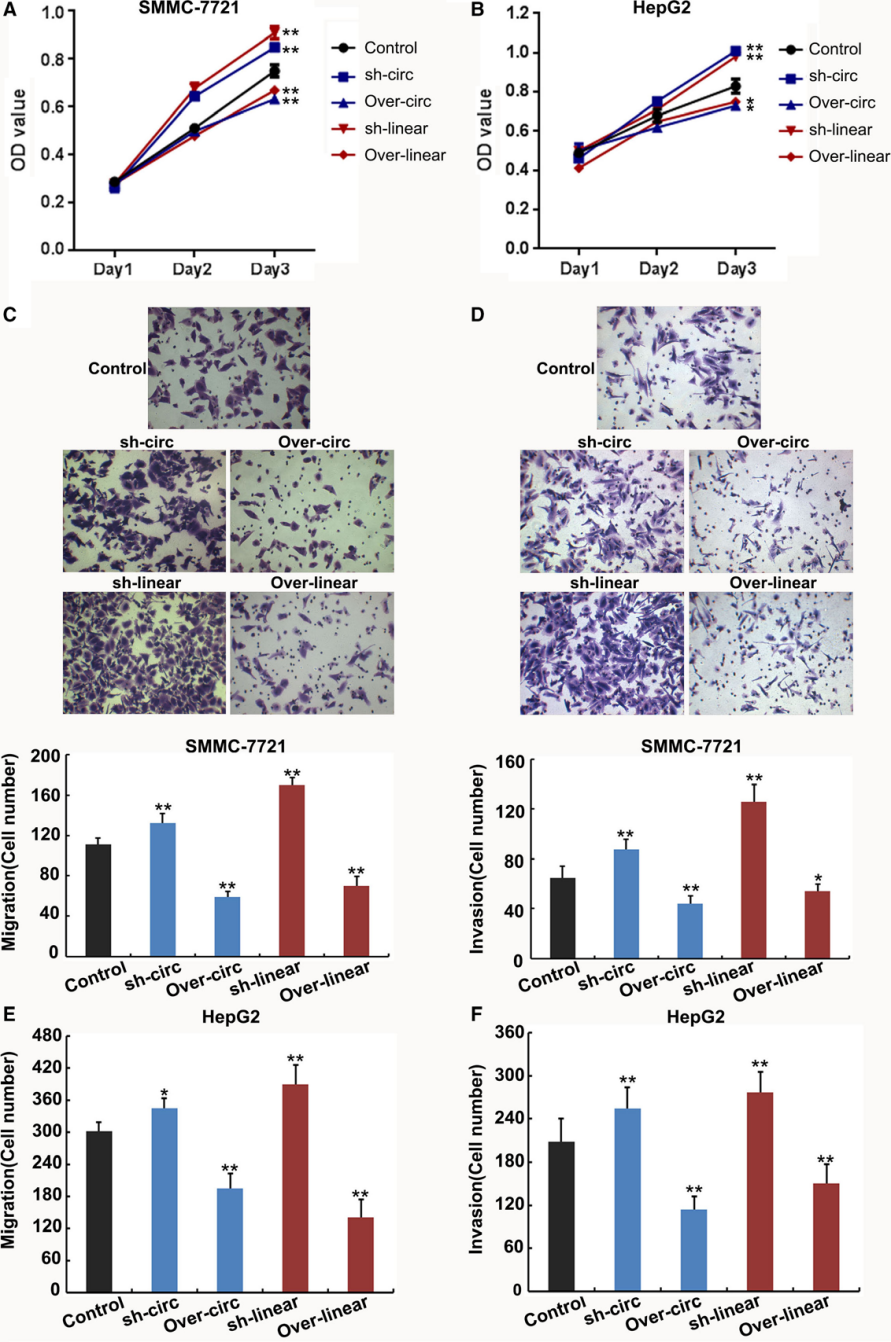
Cell proliferation, invasion, and migration of the SMMC‐7721 cell line and HepG2 cell line following the overexpression and knockdown of *circZKSCAN1* or *ZKSCAN1*. (A) MTS method examining SMMC‐7721 and HepG2 (B) cell proliferation of five different treatments on days 1, 2, and 3. (C) Migration assay and invasion assay (D) of SMMC‐7721 cells. Migration assay (E) and invasion assay (F) of HepG2 cells transfected with different treatments. Images are representative of the cells invading one field. **P <* *0.001. Control: negative control; sh‐circ: knockdown treatment of *circZKSCAN1*; over‐circ: overexpression treatment of *circZKSCAN1*; sh‐linear: knockdown treatment of *ZKSCAN1*; over‐linear: overexpression treatment of *ZKSCAN1*.

To test whether *ZKSCAN1* mRNA and *cirZKSCAN1* could affect the biological behavior of HCC cells, we performed gain‐of function and loss‐of‐function assays in SMMC‐7721 cells and HepG2 cells. We assessed the effects of the two RNA on the proliferation of HCC cells. The results of the MTS assays revealed increased proliferative ability in *ZKSCAN1* mRNA and *cirZKSCAN1* SMMC‐7721‐knockdown cells, while a sharp reduction in the proliferation rate was observed in SMMC‐7721‐overexpressing cells (Fig. 4A). The same conditions were detected in HepG2 cells (Fig. 4B). The results suggested that knockdown of *ZKSCAN1* or *cirZKSCAN1* could effectively accelerate cell proliferation. In contrast, overexpression of ZKSCAN1 or cirZKSCAN1 effectively inhibited cell proliferation. The Boyden chamber and migration through a polycarbonate membrane showed that overexpression of *ZKSCAN1* or *cirZKSCAN1* reduced cell migration (Fig. 4C,E) and cell invasion (Fig. 4D,F). In contrast, suppression of ZKSCAN1 or cirZKSCAN1 had the reverse effect on invasion (Fig. 4C,E) and cell migration (Fig. 4D,F). These experiments showed that overexpression and knockdown in SMMC‐7721 cells provided more significant results, and thus, SMMC‐7721‐based cell lines were used in subsequent studies.

### 3.5 Overexpression and knockdown of *ZKSCAN1* or *cirZKSCAN1* modulated tumor growth *in vivo*

**Figure 5:**
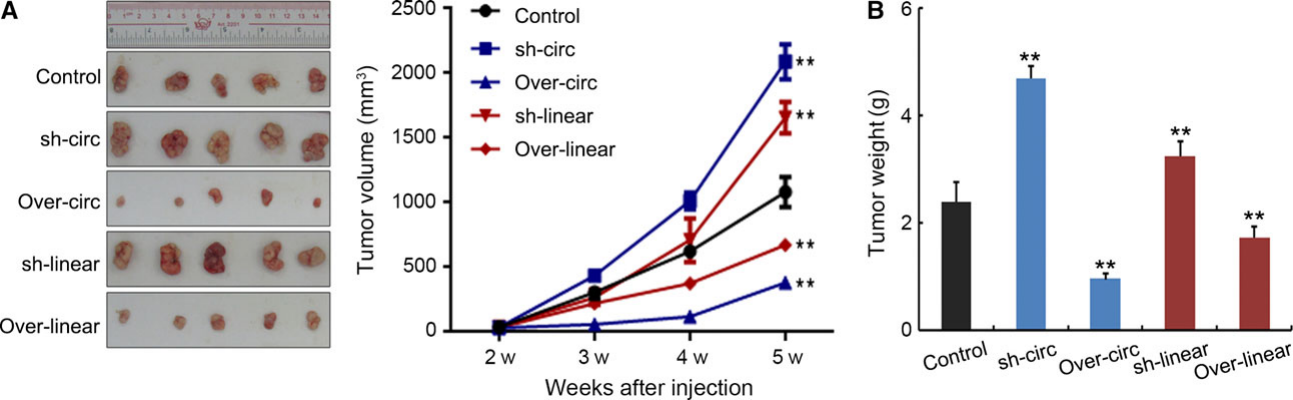
Liver xenografts in each group at 5 weeks after subcutaneous implantation of five different SMMC‐7721 cells. The mean tumor volumes and weight in five nude mice in each group are shown at different time points (right). **P <* *0.001 compared with the controls. Control: negative control; sh‐circ: knockdown treatment of *circZKSCAN1*; over‐circ: overexpression treatment of *circZKSCAN1*; sh‐linear: knockdown treatment of *ZKSCAN1*; over‐linear: overexpression treatment of *ZKSCAN1*.

As shown in Fig. 5, SMMC‐7721 cells with overexpressed *ZKSCAN1* or *cirZKSCAN1* were subcutaneously injected into the back flank of nude mice. Our results showed that the growth of tumors from overexpressed *ZKSCAN1* or *cirZKSCAN1* xenografts was significantly inhibited compared with that from the control xenografts. In contrast, the growth of tumors from knockdown *ZKSCAN1* or *cirZKSCAN1* xenografts was significantly accelerated compared with that from the control xenografts.

### 3.6 Cellular localization

**Figure 6:**
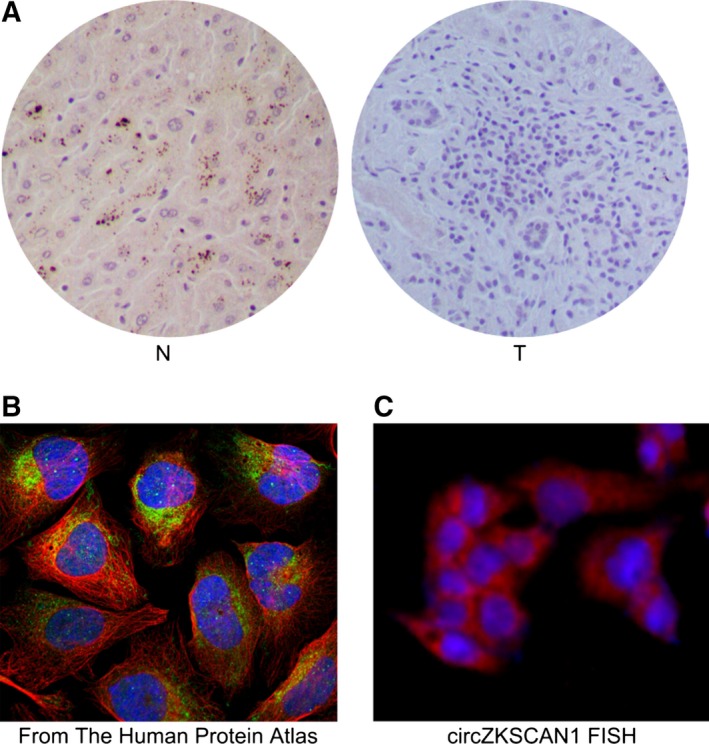
Cellular localization of *ZKSCAN1* and *circZKSCAN1*. (A) Immunohistochemistry analysis of *ZKSCAN1* in the normal (N) and HCC tissues (T). (B) FISH analysis of *circZKSCAN1* cellular localization in the Human Protein Atlas Database (http://www.proteinatlas.org/). (C) FISH analysis of the cellular localization of *circZKSCAN1* in this study.

The function of a gene is closely related to its intracellular localization. As a member of the zinc finger protein family, ZKSCAN1 protein has been reported to be located in the nucleus of gastric cancer tissues and to play regulatory roles affecting a variety of genes at the transcriptional level (Fan *et al*., 2015). Immunohistochemistry analysis using an anti‐human ZKSCAN1 antibody was used to examine its protein expression level and cellular localization in the normal and HCC liver tissues. Surprisingly, our results revealed relatively stronger, discrete, and granular cytoplasmic staining in the normal liver samples (Fig. 6A). In contrast, little *ZKSCAN1* was expressed in HCC liver tissues. In addition, information concerning the location of *ZKSCAN1* in the human protein atlas (http://www.proteinatlas.org/) showed that the majority of malignant gliomas, prostate and colorectal cancers, as well as most other malignancies, showed moderate to strong staining of *ZKSCAN1*, which was located in the nucleus, but only weak or negative cytoplasmic staining in liver cancer, which was consistent with our results. Furthermore, data from the database mentioned above showed that *ZKSCAN1* was most likely located in mitochondria in certain cell lines, which indicated that *ZKSCAN1* may play a transcription‐independent role in mitochondria in HCC. Circular RNA with different origins may be located in different parts of the cell, with circRNA originating from exons located mainly in the cytoplasm while those originating from introns are located in the nucleus. FISH analysis showed that *circZKSCAN1* was most likely located in the cytoplasm (Fig. 6B,C), consistent with previous studies (Liang and Wilusz, 2014), and it might be involved in the regulatory network of the cell.

### 3.7 RNA‐seq revealed a different molecular basis for *ZKSCAN1* mRNA and *circZKSCAN1* in SMMC‐7721 cells

**Figure 7:**
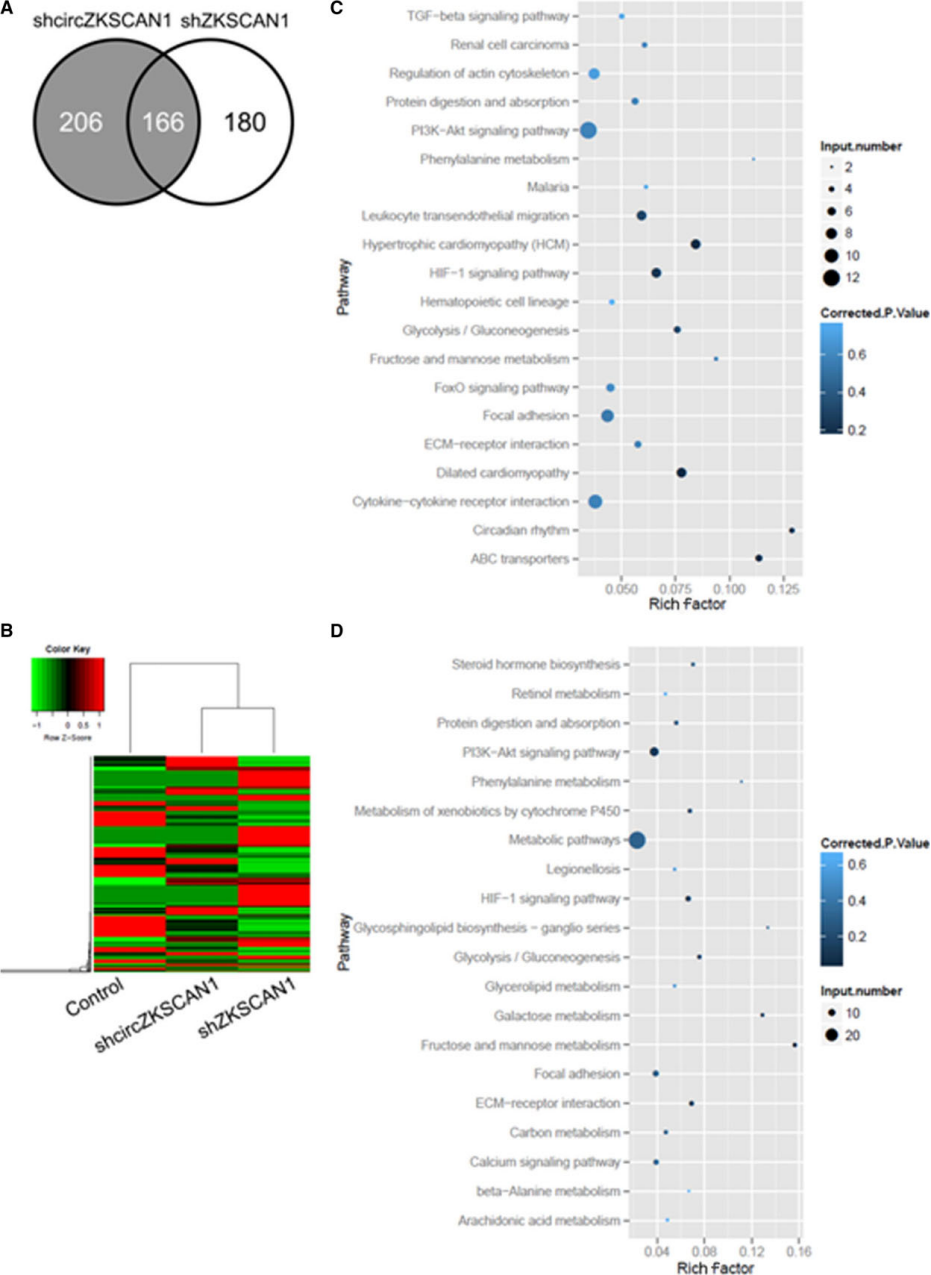
Differentially expressed genes in SMMC‐7721 cells after knockdown of *circZKSCAN1* and *ZKSCAN1*. (A) Venn diagram of the number of differentially expressed genes identified in this study. (B) Heat map analysis of the number of differentially expressed genes identified in this study. Red indicates up‐regulated expression; green indicates down‐regulated expression. Scatter diagram of the enriched KEGG pathways in (C) knockdown of *circZKSCAN1* and (D) *ZKSCAN1* cells showing differentially expressed genes. The degree of enrichment was measured according to the Rich factor, *Q*‐value, and the number of genes that were enriched in one pathway. The Rich factor is the ratio between the number of differentially expressed genes enriched in one pathway and the number of GO annotations. The greater the value of the Rich factor, the higher the degree of enrichment. The *Q*‐value is a variant of the *P*‐value, for which lower numbers equate to significant enrichment. The *y*‐axis shows the name of the pathway, and the *x*‐axis shows the Rich factor. The point size indicates the number of differentially expressed genes in one pathway, and the color of the point denotes the range of the *Q*‐value.

To explore the underlying mechanism by which *ZKSCAN1* regulated cell proliferation and invasion in HCC cells, we performed RNA‐seq analysis to identify changes in gene expression after knockdown of *ZKSCAN1* mRNA (*shZKSCAN1*) or *circZKSCAN1* (*shcircZKSCAN1*) in SMMC‐7721 cells. We detected 372 differentially expressed genes in SMMC‐7721‐knockdown cells (*shcirZKSCAN1*) compared with the negative control SMMC‐7721 cells (control). Additionally, there were 346 differentially expressed genes in SMMC‐7721 cells following the knockdown of *ZKSCAN1*. There were 166 overlapping differentially expressed genes in both of the sets mentioned above, which suggested that there were significant differences between the *shZKSCAN1* and *shcirZKSCAN1* treatments (Fig. 7A). Moreover, the genes that were significantly differentially expressed were also captured in the heat map (Fig. 7B). To further assess the biological insight gleaned from the transcript‐level response to the loss of *circZKSCAN1*, we performed KEGG enrichment analysis. The differentially expressed genes were more likely to be enriched in the PI3K pathway, migration pathway, actin cytoskeleton pathway, adhesion pathway, and cytokine interaction pathway, among others, following the knockdown of *cirZKSCAN1* (Fig. 7C). However, the differentially expressed genes following the knockdown of *ZKSCAN1* were mainly enriched in metabolic pathways (Fig. 7D).

### 3.8 qRT‐PCR analysis of *ZKSCAN1‐* and *cirZKSCAN1*‐related downstream genes in SMMC‐7721 cells

**Figure 8:**
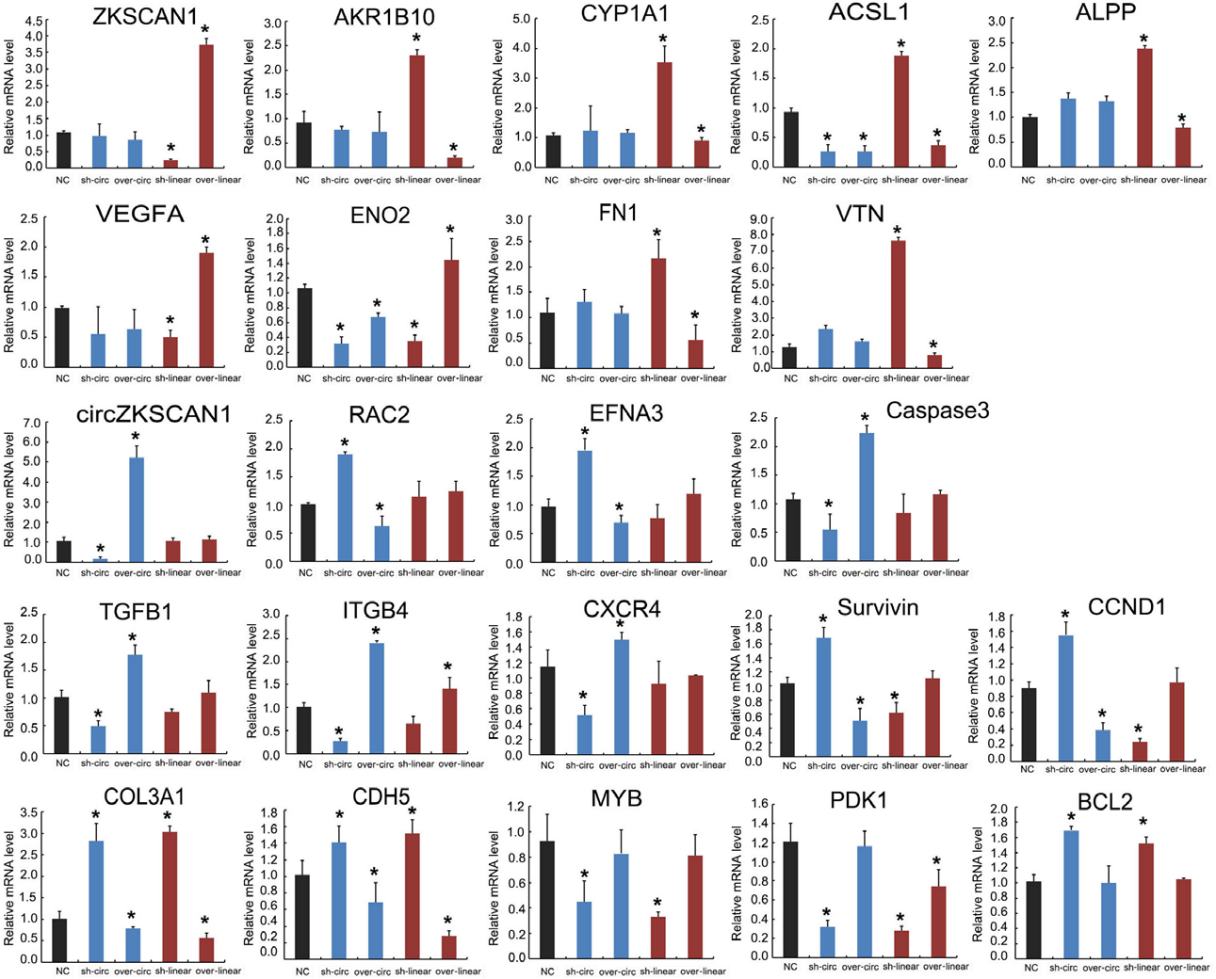
qRT‐PCR analysis of the expression of 21 genes selected from the RNA‐seq results. Control: normal SMMC‐7721 cell line; sh‐circ: knockdown treatment of circZKSCAN1 in the SMMC‐7721 cell line; over‐circ: overexpression treatment of circZKSCAN1 in the SMMC‐7721 cell line; sh‐linear: knockdown treatment of ZKSCAN1 in the SMMC‐7721 cell line; over‐linear: overexpression treatment of ZKSCAN1 in the SMMC‐7721 cell line. The *y*‐axis shows the gene expression levels after normalization to the reference gene GAPDH. *P <0.05, compared with NC.

We validated the *ZKSCAN1‐* and *cirZKSCAN1*‐related downstream genes in SMMC‐7721 cells by qRT‐PCR analysis (Fig. 8). AKR1B10, CYP1A1, ACSL1, and ALPP, which were related to cellular metabolism, were synchronously up‐/down‐regulated with the knockdown and overexpression of *ZKSCAN1*, but not *cirZKSCAN1*. Similar expression patterns were detected in apoptosis and migration‐related genes, including VEGFA, ENO2, FN1, and VTN. The expression levels of RAC2, EFNA3, and caspase 3, which are important factors in the intrinsic apoptosis pathway, were only affected by knockdown and overexpression treatments of *cirZKSCAN1*. Similar expression patterns were detected in cell proliferation‐related genes, including TGFB1, ITGB4, CXCR4, survivin, and CCND1. However, there was also an interaction between *cirZKSCAN1* and *ZKSCAN1*, and the expression levels of COL3A1, CDH5, MYB, PDK1, and BCL2 were regulated by both *cirZKSCAN1* and *ZKSCAN1*.

The above results clearly indicated that the two mature RNA transcripts from the same ZKSCAN1 gene may play different roles in HCC cells. We propose that after translation, ZKSCAN1 protein is translocated to mitochondria and plays a regulatory role in metabolism, further impacting cell death and metastasis. *circZKSCAN1* may function as a ceRNA, which is regulated by other RNA by competing for the same pool of miRNA, playing a role in proliferation‐ and invasion‐related signaling pathways. The detailed regulatory mechanism requires further investigation.

## 4 Discussion

*ZKSCAN1* had been reported to be up‐regulated in gastric cancer (Bartel, 2009). However, few reports have examined the role of *ZKSCAN1* in HCC, and little is known about its molecular basis. Through a series of functional experiments in the present study, we showed that, in addition to *ZKSCAN1* mRNA, a circular RNA, c*irZKSCAN1*, was present in HCC tissues and cells. The expression levels of *cirZKSCAN1* and *ZKSCAN1* were both down‐regulated in HCC cell lines. However, there was no obvious relationship between *ZKSCAN1* and *cirZKSCAN1* expression. The potential diagnostic analysis suggested that *cirZKSCAN1* had the maximum value compared with *ZKSCAN1*. Knockdown treatment of *ZKSCAN1* or *cirZKSCAN1* effectively accelerated cell proliferation, migration, and invasion. In contrast, overexpression of *ZKSCAN1* or *cirZKSCAN1* effectively inhibited cell proliferation, migration, and invasion. Additionally, the growth of tumors from knockdown *ZKSCAN1* or *cirZKSCAN1* xenografts was significantly accelerated compared with the control xenografts. *ZKSCAN1*, as a member of the zinc finger protein family, is a transcription factor and thus theoretically was more likely to be located in the nucleus. However, most of the ZKSCAN1 protein was located in the cytoplasm, and more often in mitochondria in the normal liver tissues. FISH analysis of the location of *cirZKSCAN1*, which was derived from exons, showed that it was most likely located in cytoplasm, which is consistent with previous results (Jeck and Sharpless, 2014). Furthermore, significant differential expression was observed in downstream genes following the knockdown of *ZKSCAN1* or *cirZKSCAN1* compared with the negative control cells in the RNA‐seq analysis. KEGG analysis suggested that these differentially expressed genes after *circZKSCAN1* silencing were more likely to be enriched in the PI3K pathway, migration pathway, actin cytoskeleton pathway, adhesion pathway, and cytokine interaction pathway, among others. However, the differentially expressed genes were enriched in metabolic pathways following the knockdown of *ZKSCAN1*. qRT‐PCR analysis suggested that the expression levels of cell metabolism, apoptosis, migration, and cell proliferation‐related genes were consistent with the RNA‐seq data. Therefore, *cirZKSCAN1* and *ZKSCAN1* could play important regulatory roles in different signaling pathways.

Based on our previous studies, *cirZKSCAN1* and *ZKSCAN1* are involved in the progression of HCC, as shown in Fig. S1. The *ZKSCAN1* gene can generate two types of RNA, including *ZKSCAN1* mRNA and *cirZKSCAN1*. *ZKSCAN1* mRNA can be translated into a functional protein, which is likely to be located in mitochondria and appears to play an important role in maintaining the normal metabolism of HCC cells. The metabolism‐facilitated proteins CYP1A1, ACSL1, ALPP, and AKR1B10 were factors downstream of *ZKSCAN1* protein. Metabolic disorders further impacted cell death and metastasis, in part by regulating FN1, VTN, ENO2, and VEGFA. Additionally, *cirZKSCAN1* may have acted as a competitive inhibitor to retain the endogenous RNA and to regulate the expression of tumor cell proliferation and metastasis‐related genes, including the apoptotic genes RAC2, EFNA3, and caspase 3, and the cell proliferation‐related genes TGFB1, ITGB4, CXCR4, survivin, and CCND1. We assumed that *cirZKSCAN1* acted as a ceRNA to regulate its circRNA–miRNA–mRNA network and that the interactions could be predicted by TargetScan, miRanda, and our RNA‐seq data. Based on these analyses, a total of eight miRNA and 87 mRNA were predicted to interact with *cirZKSCAN1* (Fig. S2). RNA pull‐down and other experiments are needed to further elucidate the mechanism of *circZKSCAN1*. Thus, the two *ZKSCAN1* gene post‐translational products (*ZKSCAN1* mRNA and circRNA) play important roles in the development of human HCC cells, acting independently and cooperating closely with one another to promote cancer growth.

## 5 Conclusions

In summary, we demonstrated the expression profiles of *cirZKSCAN1* and *ZKSCAN1* in the human HCC tissues and cell lines. *CirZKSCAN1* had greater potential value for the diagnosis of HCC compared with *ZKSCAN1*. Furthermore, overexpression and knockdown treatments of *cirZKSCAN1* and *ZKSCAN1* effectively influenced the proliferation, invasion, and migration of human HCC cells. In addition, ZKSCAN1 protein was located in the cytoplasm, especially in the mitochondria in the normal liver tissues. As an exonic circRNA, *cirZKSCAN1* was located in the cytoplasm. The downstream signaling pathways were significantly differentially expressed following the knockdown of *ZKSCAN1* or *cirZKSCAN1* compared with the negative control cells in the RNA‐seq analysis. A putative pathway for *cirZKSCAN1* and *ZKSCAN1* suggested that the *ZKSCAN1* gene could be involved in the metabolism, apoptosis, proliferation, and metastasis of HCC. The results presented herein suggest that *cirZKSCAN1* may function as a diagnostic biomarker of HCC and may provide a novel understanding of the mechanisms associated with human HCC.

## Author contributions

ZY, JL, BL, and YY conceived and designed the project; KH, JL, and HH acquired the data; QW, PZ, ZX, CH, and ZH analyzed and interpreted the data; ZY and JL wrote the manuscript.

## Supporting information


**Fig. S1.** The potential molecular mechanism of *ZKSCAN1* and *circZKSCAN1* in the progression of HCC cancer growth.

**Fig. S2.** The predicted *circZKSCAN1*‐targeted *circRNA*–miRNA–mRNA/gene network based on the RNA‐seq data.
