# Corrigendum to: ZKSCAN1 gene and its related circular RNA (circZKSCAN1) both inhibit hepatocellular carcinoma cell growth, migration, and invasion but through different signaling pathways

**DOI:** 10.1002/1878-0261.12578

**Published:** 2019-10-07

In the paper by Yao *et al. *(2017), it was originally stated in the Acknowledgements that the study was funded by the National Natural Science Foundation of China (8157111144, 81570593). This is incorrect and should have read ‘This project was funded by the National Natural Science Foundation of China (81572726, 81570593)’.

In addition, the article contained multiple instances of the following typo: circZKSCAN1 was incorrectly written as cirZKSCAN1. The correct spelling in all cases is circZKSCAN1.
