# Supplementary figures and images for: ZKSCAN1 gene and its related circular RNA (circZKSCAN1) both inhibit hepatocellular carcinoma cell growth, migration, and invasion but through different signaling pathways
Source: Mol Oncol. 2017 Mar 17;11(4):422–37. doi: 10.1002/1878-0261.12045 (PMC5527481; doi:10.1002/1878-0261.12045)

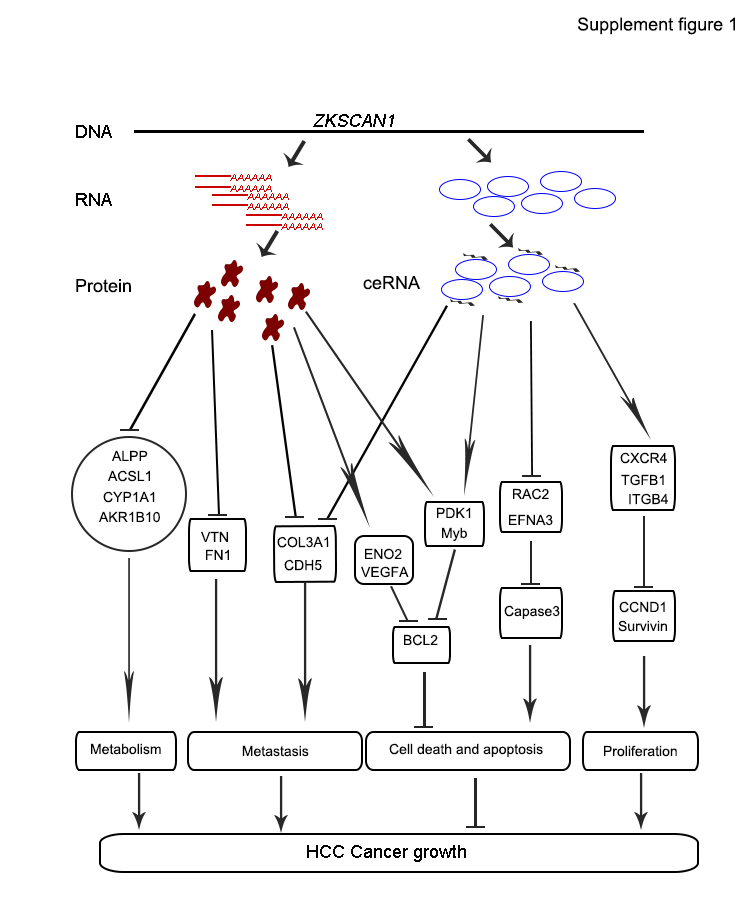

Supplement: Supplementary file 1 — Fig. S1. The potential molecular mechanism of ZKSCAN1 and circZKSCAN1 in the progression of HCC cancer growth. [file MOL2-11-422-s001.tiff]

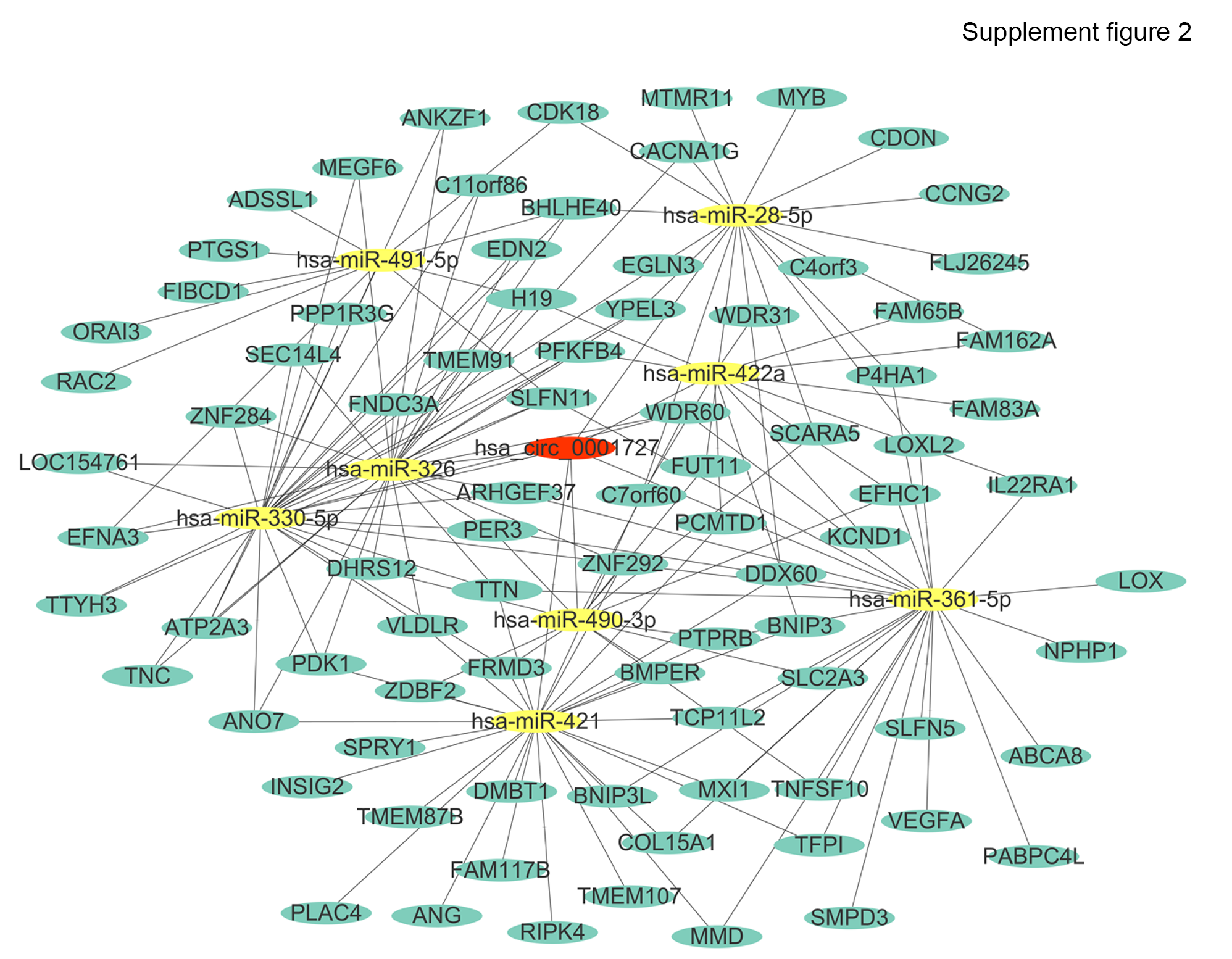

Supplement: Supplementary file 2 — Fig. S2. The predicted circZKSCAN1‐targeted circRNA–miRNA–mRNA/gene network based on the RNA‐seq data. [file MOL2-11-422-s002.tiff]
